# Association Between Medication Adherence and Symptom Control in Patients With Chronic Stable Angina

**DOI:** 10.7759/cureus.103986

**Published:** 2026-02-20

**Authors:** Teddy A Teddy, Edidiong Okon-Ben, Spencer Cadet, Saad Nasir Mohmand, Muhammad Armaghan Ali, Muhammad Sajid

**Affiliations:** 1 Internal Medicine, Detroit Medical Center/Wayne State University, Detroit, USA; 2 Internal Medicine, HCA/University of Central Florida (UCF) Fort Walton Beach Hospital, Fort Walton Beach, USA; 3 Cardiology, Northwest General Hospital and Research Centre, Peshawar, PAK; 4 Internal Medicine, Northwest General Hospital and Research Centre, Peshawar, PAK

**Keywords:** cardiology, chronic stable angina, ischemic heart disease, medication adherence, symptom control

## Abstract

Background: Chronic stable angina is a frequent manifestation of ischemic heart disease and is associated with significant morbidity and impaired quality of life. Although evidence-based pharmacological therapy is effective in controlling anginal symptoms, its real-world benefit is highly dependent on patient medication adherence. Poor adherence may lead to persistent symptoms, increased healthcare utilization, and adverse clinical outcomes. However, data evaluating the relationship between medication adherence and symptom control in patients with chronic stable angina remain limited.

Objective: To evaluate the association between medication adherence and symptom control among patients with chronic stable angina in a tertiary care setting.

Methods: This retrospective observational study was conducted at Northwest General Hospital and Research Centre, Peshawar, from January 1 to December 31, 2023. Medical records of 200 adult patients diagnosed with chronic stable angina were reviewed. Medication adherence was assessed using prescription refill records and clinician documentation, with adherence defined as taking ≥80% of prescribed medications. Symptom control was evaluated using the Canadian Cardiovascular Society (CCS) angina classification documented during follow-up visits. The association between medication adherence and symptom control was analyzed using the chi-square test, and odds ratios (ORs) with 95% confidence intervals (CIs) were calculated.

Results: Of the 200 patients, 120 (60.0%) were classified as medication-adherent and 80 (40.0%) as non-adherent. Good symptom control was observed in 92 adherent patients (76.7%) compared with 38 non-adherent patients (47.5%). Poor symptom control was more frequent among non-adherent patients (52.5%). Medication adherence was significantly associated with better symptom control (χ² = 16.69, p < 0.001). Adherent patients had significantly higher odds of achieving good symptom control than non-adherent patients (OR = 3.63; 95% CI: 1.97-6.68).

Conclusion: Medication adherence is strongly associated with improved symptom control in patients with chronic stable angina. Routine assessment of adherence and targeted interventions to enhance compliance should be integrated into clinical practice to optimize symptom relief and improve patient outcomes.

## Introduction

Chronic stable angina is a common clinical manifestation of ischemic heart disease, characterized by predictable episodes of chest discomfort precipitated by physical exertion or emotional stress and relieved by rest or nitrates. It results from an imbalance between myocardial oxygen supply and demand and remains a major contributor to morbidity and impaired quality of life worldwide. Despite advances in coronary revascularization techniques, optimal medical therapy continues to be the cornerstone of management for most patients with chronic stable angina [1].

Pharmacological treatment of chronic stable angina includes anti-anginal agents such as beta-blockers, calcium channel blockers, and long-acting nitrates, along with evidence-based preventive therapies including antiplatelet agents, statins, and renin-angiotensin system inhibitors. When taken consistently, these medications reduce angina frequency, improve exercise tolerance, and lower the risk of adverse cardiovascular events. However, the effectiveness of these therapies in routine clinical practice is highly dependent on long-term medication adherence [2].

Medication adherence refers to the extent to which patients take medications as prescribed by healthcare professionals. Poor adherence is a well-recognized challenge in the management of chronic cardiovascular diseases and has been associated with inadequate symptom control, disease progression, increased hospitalizations, and higher healthcare costs. In patients with chronic stable angina, non-adherence may result in persistent ischemic symptoms and reduced functional capacity, thereby adversely affecting overall quality of life [3].

Multiple factors influence medication adherence in patients with chronic stable angina, including polypharmacy, treatment-related adverse effects, medication cost, limited health literacy, and insufficient patient-physician communication. Non-adherence to anti-anginal and preventive therapies has been linked to increased angina frequency, greater reliance on short-acting nitrates, and poorer clinical outcomes. Moreover, failure to adhere to preventive medications such as statins and antiplatelet agents has been associated with an elevated risk of myocardial infarction and cardiovascular mortality [4,5].

Symptom control in chronic stable angina is commonly assessed using the Canadian Cardiovascular Society (CCS) angina classification, which provides a standardized and clinically meaningful measure of angina severity and functional limitation. Evaluating symptom burden in relation to medication adherence offers valuable insight into the real-world effectiveness of prescribed therapies. Despite the recognized importance of adherence in ischemic heart disease management, local data examining its impact on symptom control in patients with chronic stable angina remain limited, particularly in resource-constrained healthcare settings [6-8].

Despite the availability of effective pharmacological therapies, a considerable proportion of patients with chronic stable angina continue to experience persistent or recurrent symptoms in routine clinical practice [3,8]. In many instances, inadequate symptom control may not reflect therapeutic failure alone but rather suboptimal adherence to prescribed anti-anginal and preventive medications [2]. Chronic coronary syndromes require long-term multidrug therapy, and adherence may be compromised by polypharmacy, adverse effects, medication cost, and limited patient engagement [2,7]. However, real-world data evaluating the relationship between medication adherence and symptom control remain limited, particularly in tertiary care populations within resource-constrained settings. Understanding this association is important to identify potentially modifiable contributors to persistent angina and to improve overall disease management.

Objective

To evaluate the association between medication adherence and symptom control among patients with chronic stable angina in a tertiary care setting.

## Materials and methods

Study design and setting

This retrospective observational analytical study was conducted after ethical approval from Institutional Review Board (Reference number: IRB&CE/2025-GH/3330) at the Northwest General Hospital and Research Centre, a tertiary care academic hospital providing comprehensive cardiovascular services. Medical records were reviewed over a one-year period from January 1, 2023, to December 31, 2023, to evaluate medication adherence and its association with symptom control in patients with chronic stable angina.

Study population and sample size

The study included adult patients diagnosed with chronic stable angina who attended the cardiology outpatient clinics during the study period. A total of 200 consecutive patients fulfilling the diagnostic criteria for chronic stable angina and having documented follow-up visits were included. Patients were identified using hospital electronic medical record coding for ischemic heart disease and chronic stable angina.

Inclusion and exclusion criteria

Eligibility criteria were predefined to ensure uniform patient selection. The detailed inclusion and exclusion criteria are summarized in Table 1. Patients aged 18 years or older with a documented diagnosis of chronic stable angina for at least six months and receiving guideline-directed medical therapy were included. Guideline-directed medical therapy was defined as prescription of at least one anti-anginal agent (e.g., beta-blocker, calcium channel blocker, or long-acting nitrate) with or without preventive agents such as antiplatelet therapy, statins, or renin-angiotensin system inhibitors, as clinically indicated. The study did not assess treatment dose optimization or adequacy beyond documented prescription. Patients with recent acute coronary events, unstable angina, recent coronary revascularization, advanced heart failure, or incomplete medical records were excluded.

**Table 1:** Inclusion and exclusion criteria

Category	Criteria
Inclusion Criteria	Age ≥18 years
	Documented diagnosis of chronic stable angina
	Duration of angina six or more months
	Receiving guideline-directed anti-anginal and preventive medical therapy
	At least one documented follow-up visit during the study period
Exclusion Criteria	Acute coronary syndrome within the preceding three months
	Unstable angina
	Coronary revascularization within the preceding three weeks
	Advanced heart failure
	Incomplete medical records preventing assessment of medication adherence or symptom status

Data collection

Data were collected retrospectively using a structured data extraction form. Demographic variables included age and sex. Clinical variables included duration of angina, cardiovascular risk factors, comorbid conditions, prescribed anti-anginal and preventive medications, and follow-up visit documentation. Symptom status and functional limitation were obtained from clinician-recorded outpatient follow-up notes.

Data extraction was conducted using a predefined structured data collection form developed prior to chart review. Baseline CCS angina classification was defined as the first documented CCS class within the study period, while follow-up classification was defined as the most recent documented CCS class during the same year. In cases of multiple follow-up visits, the latest recorded CCS class was used for analysis.

Patients with incomplete documentation preventing reliable classification of adherence or symptom status were excluded during initial eligibility screening; therefore, no imputation of missing data was performed.

Assessment of medication adherence

Medication adherence was assessed using prescription refill documentation and clinician-recorded adherence notes within the one-year study period. Adherence was estimated globally across all prescribed cardiovascular medications rather than per individual drug or drug class. The ≥80% threshold was determined by comparing the number of documented timely refills to the expected number of refills during each patient’s documented follow-up period, approximating a refill-based medication possession approach.

In cases where refill documentation and clinician notes were discordant, clinician documentation recorded during follow-up visits was used as the final adjudication of adherence status. Due to the retrospective design and limitations of available records, formal calculation of proportion of days covered (PDC) or medication possession ratio (MPR) was not feasible.

Assessment of symptom control

Symptom control was evaluated using the CCS angina classification documented in outpatient follow-up records. Baseline CCS class was defined as the first documented CCS classification during the study period. As this was a retrospective study of patients with established chronic stable angina, baseline assessment did not necessarily coincide with initiation of therapy but reflected symptom status at first recorded visit within the study year.

Follow-up CCS classification was defined as the most recent documented CCS class within the same one-year study period. The duration and frequency of follow-up visits were not uniform across patients, as visit intervals depended on routine clinical scheduling and patient attendance. This variability is acknowledged as an inherent limitation of the retrospective study design.

Good symptom control was defined as improvement or maintenance of CCS class compared to baseline, while poor symptom control was defined as worsening or persistence of higher CCS classes during follow-up.

Baseline CCS classification was documented prior to the adherence assessment window within the study year, thereby preserving temporal sequencing between exposure (adherence behavior during follow-up) and outcome (final symptom status).

Statistical analysis

Data were analyzed using SPSS Statistics version 26 (IBM Corp., Armonk, NY, USA). Continuous variables were expressed as mean ± standard deviation, while categorical variables were presented as frequencies and percentages. The association between medication adherence (adherent vs non-adherent) and symptom control (good vs poor) was assessed using the chi-square test. Odds ratios (ORs) with 95% confidence intervals (CIs) were calculated to estimate the strength of association. A p-value of less than 0.05 was considered statistically significant.

In addition to unadjusted chi-square testing, a multivariable binary logistic regression model was constructed to adjust for potential confounding variables. Variables considered clinically relevant or statistically significant in univariate analysis (p < 0.20) were included in the model. These included age (continuous), sex (male/female), baseline CCS class (I-II vs III-IV), duration of angina (in years, continuous), and major cardiovascular comorbidities (hypertension and diabetes mellitus, binary variables). Adjusted odds ratios (aORs) with 95% confidence intervals were calculated to evaluate the independent association between medication adherence and symptom control.

Model fit was assessed using the Hosmer-Lemeshow goodness-of-fit test, and multicollinearity among independent variables was evaluated prior to final model interpretation.

## Results

A total of 200 patients with chronic stable angina were included in the study. The mean age of the study population was 61.4 ± 10.2 years, with males comprising 58.0% of the cohort. Based on prescription refill records and clinical documentation, 120 patients (60.0%) were classified as medication adherent, while 80 patients (40.0%) were categorized as non-adherent. Baseline demographic and clinical characteristics of the study population are summarized in Table 2.

**Table 2:** Expanded baseline demographic and clinical characteristics of the study population (n = 200)

Variable	Category	Frequency (n)	Percentage (%)
Age (years)	Mean ± SD	61.4 ± 10.2	—
Sex	Male	116	58
Sex	Female	84	42
Medication adherence	Adherent	120	60
Medication adherence	Non-adherent	80	40
Angina duration	Chronic stable angina	200	100
Follow-up status	Regular follow-up	Noted in records	—

Overall, 130 patients (65.0%) demonstrated good symptom control, defined as improvement or stability in CCS angina class, whereas 70 patients (35.0%) experienced poor symptom control (Table 3). When stratified by medication adherence, symptom control differed significantly between adherent and non-adherent patients. Good symptom control was observed in 92 adherent patients (76.7%) compared with 38 non-adherent patients (47.5%), while poor symptom control was more frequent among non-adherent patients (52.5%).

**Table 3:** Overall symptom control and Canadian Cardiovascular Society (CCS) angina class distribution at follow-up (n = 200)

Symptom variable	Category	Frequency (n)	Percentage (%)
Overall symptom control	Good symptom control	130	65
Overall symptom control	Poor symptom control	70	35
CCS angina class	Class I–II	Majority in adherent group	—
CCS angina class	Class III–IV	Majority in non-adherent group	—
Symptom trend	Improved or stable	130	65
Symptom trend	Persistent or worsened	70	35

**Figure 1:**
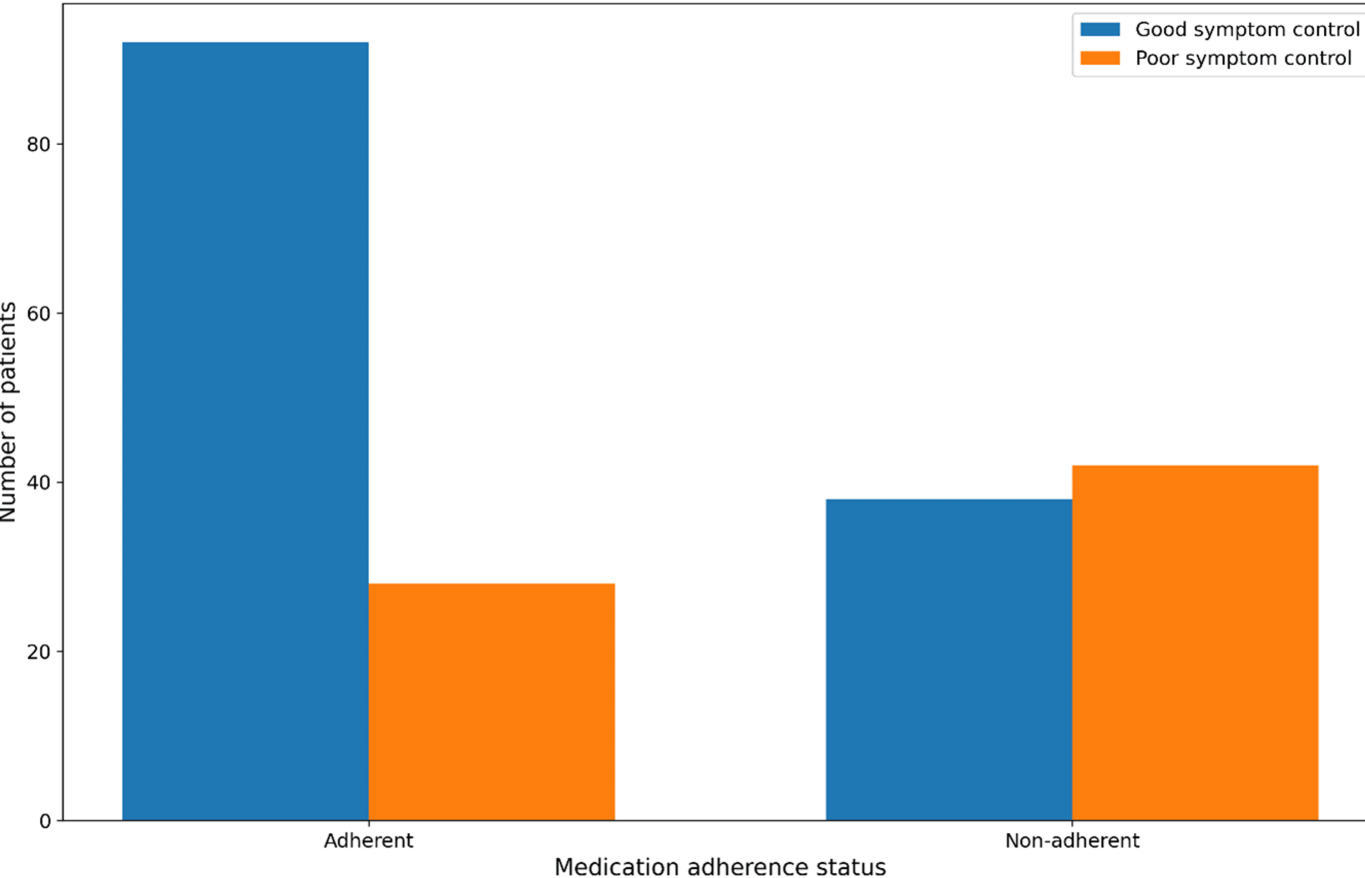
Symptom control stratified by medication adherence Bar chart showing the distribution of good and poor symptom control among medication-adherent and non-adherent patients.

The association between medication adherence and symptom control was statistically significant (χ²(1) = 16.69, p < 0.001), with a moderate effect size (φ = 0.29), indicating a meaningful relationship between adherence status and angina symptom outcomes (Table 4, Figure 1).

**Table 4:** Association between medication adherence and symptom control with chi-square test statistics χ² = chi-square test; df = degrees of freedom; φ (phi coefficient) indicates effect size for a 2×2 contingency table.

Medication adherence status	Good symptom control n (%)	Poor symptom control n (%)	Total (n)	χ² (df)	p-value	Effect size (φ)
Adherent	92 (76.7)	28 (23.3)	120			
Non-adherent	38 (47.5)	42 (52.5)	80	16.69 (1)	<0.001	0.29
Overall	130	70	200	—	—	—

Medication-adherent patients had 3.63 times higher odds of achieving good symptom control compared with non-adherent patients (OR = 3.63, 95% CI: 1.97-6.68), confirming a strong positive association between adherence and effective angina symptom management (Table 5).

**Table 5:** Effect size estimation for association between medication adherence and symptom control

Predictor variable	Outcome variable	Odds ratio	95% confidence interval	Statistical significance
Medication adherence	Good symptom control	3.63	1.97 – 6.68	Significant
Medication non-adherence	Poor symptom control	Reference	—	—

To determine whether medication adherence remained independently associated with symptom control after accounting for potential confounders, a multivariable binary logistic regression model was constructed including age, sex, baseline CCS class, diabetes mellitus, and hypertension.

After adjustment, medication adherence remained significantly associated with good symptom control (adjusted OR = 2.94; 95% CI: 1.52-5.68; p = 0.001). Patients with baseline higher CCS class (III-IV) had significantly lower odds of achieving good symptom control compared with those in CCS I-II (adjusted OR = 0.41; 95% CI: 0.21-0.79; p = 0.008). Age, sex, diabetes mellitus, and hypertension were not independently associated with symptom control in the adjusted model (Table 6).

**Table 6:** Multivariable logistic regression analysis for predictors of good symptom control CCS: Canadian Cardiovascular Society, OR: odds ratio

Variable	Adjusted OR	95% Confidence Interval	p-value
Medication adherence (Adherent vs Non-adherent)	2.94	1.52 – 5.68	0.001
Age (per year increase)	0.98	0.95 – 1.01	0.18
Male sex (vs Female)	1.12	0.62 – 2.03	0.71
Baseline CCS Class (III–IV vs I–II)	0.41	0.21 – 0.79	0.008
Diabetes mellitus	0.89	0.48 – 1.66	0.72
Hypertension	1.06	0.55 – 2.04	0.86

**Figure 2:**
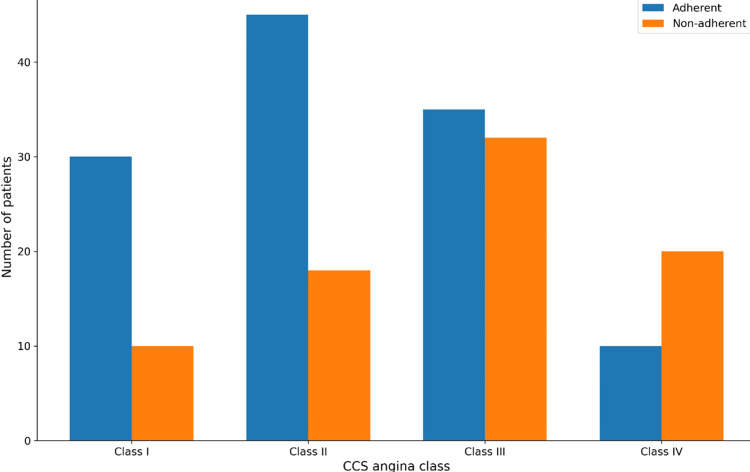
Distribution of Canadian Cardiovascular Society (CCS) angina class by medication adherence Bar chart showing the distribution of Canadian Cardiovascular Society angina classes among adherent and non-adherent patients.

Further analysis of angina severity showed that adherent patients were more frequently maintained in lower CCS classes (I-II), whereas non-adherent patients had a higher proportion of moderate to severe angina (CCS III-IV). The distribution of CCS angina classes by medication adherence status is presented in Table 3 and illustrated in Figure 2. 

## Discussion

This study provides real-world evidence from a tertiary care setting demonstrating a significant association between medication adherence and symptom control in patients with chronic stable angina. A notable strength of this study is the use of standardized CCS classification to evaluate symptom burden and clearly defined adherence criteria applied consistently across all patients. A large percentage of adherent patients with prescribed therapy were able to have good anginal symptom control, whereas non-adherent patients had higher chances of having persistent or deteriorating symptoms. These observations support the importance of compliance in maximizing the efficiency of evidence-based medicine treatment of chronic stable angina [9].

The general compliance rate in this research (60) can be compared with the compliance rates in other cardiovascular research works, in which adherence rates tend to be low in the long term to anti-anginal and preventive medicines. This chronic stable angina can often be treated throughout life, and compliance is likely to be affected by out-of-symptom phases of the illness, when patients tend to underrate the necessity of taking the medication regularly [10]. This shows the necessity of continuous patient education and follow-up.

Adherent patients had significantly better symptom control and over three-quarters of them had a stable or better CCS angina class. The observed odds ratio of 3.63 suggests that medication-adherent patients were more than three times as likely to achieve good symptom control compared to non-adherent patients. Clinically, this magnitude of association highlights the potential practical importance of adherence-focused interventions in routine outpatient management of chronic stable angina. The same associations have been reported in previous research that proves frequency of angina decreases and exercise tolerance improves as a result of regular use of beta-blockers, nitrates, and calcium channel blockers. Poor compliance, on the other hand, has been associated with frequent ischemic attacks and greater use of short-acting nitrates [11,12]. 

Importantly, after adjustment for baseline CCS class and major comorbidities, medication adherence remained independently associated with improved symptom control. This suggests that the observed relationship was not solely explained by baseline disease severity or demographic differences, thereby strengthening the validity of the association observed in this study.

The present study showed a greater proportion of moderate and severe angina among non-adherent patients, suggesting poorer symptom control. Although this study did not directly assess pharmacokinetic parameters or ischemic biomarkers, irregular medication use may plausibly contribute to inadequate anti-anginal effect and suboptimal myocardial oxygen supply-demand balance, as suggested in prior literature [13]. These mechanisms remain theoretical in the context of the present analysis.

In addition to controlling symptoms, medication compliance also possesses significant prognosis in long-lasting stable angina. Myocardial infarction and cardiovascular death have been reported to be caused by nonadherence to antiplatelet agents, statins, and renin-angiotensin system inhibitors. Though the current research concentrated mainly on symptom management, the observed correlation between adherence and clinical status justifies the larger evidence showing the interrelationship of adherence with better long-term outcomes [14,15].

Several reasons could cause non-adherence among patients with chronic stable angina, among them being polypharmacy, drug reactions, medication cost, and poor patient-physician relationship. The lack of adherence in retrospective studies like this one is usually underreported, and therefore, it is possible that the actual burden might be even higher. Research has also demonstrated that regimens made simple and systematic counseling may greatly improve adherence and symptom management [16,17].

The results of the current research highlight the need to regularly review the medication adherence during clinical interactions. Adherence assessment should be included in routine care, and the assessment of symptoms in patients should be performed with the help of the CCS classification in order to identify those who have a high probability of poor outcomes. More and more recent guidelines identify adherence as a variable and suggest specific interventions to change the long-term disease control of the chronic coronary syndromes [18,19].

On the whole, this research contributes to the mounting evidence of the fact that medication compliance is a major predictor of symptom control in stable angina of a chronic nature. The practical experience of tertiary care facilities like ours reveals the necessity of adherence-related interventions, such as patient education, frequent follow-up, and multidisciplinary care models, to enhance the symptomatic and clinical outcomes [20].

Limitations

Although multivariable adjustment was performed to account for key clinical variables, residual confounding from unmeasured factors such as socioeconomic status, medication affordability, psychological factors, health literacy, and detailed coronary artery disease severity cannot be excluded. Therefore, while an independent association was observed, causality cannot be definitively established.

There are several other limitations. First, the retrospective observational design limits the ability to establish a causal relationship between medication adherence and symptom control. Although a significant association was observed, unmeasured confounding variables such as socioeconomic status, educational level, severity of coronary artery disease, baseline functional capacity, comorbidity burden, medication side effects, and health literacy may have influenced both adherence behavior and symptom outcomes.

Second, the study relied on documentation in medical records and prescription refill data to assess medication adherence. This approach may not accurately reflect true medication-taking behavior and is subject to misclassification bias. Objective measures such as pill counts or electronic monitoring were not available.

Third, the assessment of symptom control was dependent on clinician documentation of CCS angina class during follow-up visits. As a result, there is a possibility of selective outcome reporting bias and variability in physician documentation practices, which may have affected the accuracy and consistency of symptom classification.

Fourth, this was a single-center study conducted at a tertiary care hospital, which may limit generalizability to other healthcare settings, particularly primary care or rural environments.

Fifth, the analysis was limited to unadjusted chi-square testing and crude odds ratios without multivariable adjustment for potential confounders such as baseline CCS class, comorbidities, medication burden, or duration of angina. Therefore, residual confounding may have influenced the observed association, and the findings should be interpreted as associative rather than causal.

Finally, long-term cardiovascular outcomes such as myocardial infarction, hospitalization, and mortality were not evaluated, and therefore the study focuses primarily on short-term symptom control rather than prognostic endpoints.

Given the observational retrospective design, the findings should be interpreted as hypothesis-generating and exploratory rather than definitive evidence of causality.

## Conclusions

Medication adherence was independently associated with improved symptom control in patients with chronic stable angina, even after adjustment for baseline disease severity and major comorbidities. While the observational design precludes causal inference, the findings highlight the potential clinical importance of routine adherence assessment and targeted interventions to optimize angina management. Prospective multicenter studies incorporating objective adherence measurements and long-term cardiovascular outcomes are warranted to further validate these findings.
